# Global QSAR models of skin sensitisers for regulatory purposes

**DOI:** 10.1186/1752-153X-4-S1-S5

**Published:** 2010-07-29

**Authors:** Qasim Chaudhry, Nadège Piclin, Jane Cotterill, Marco Pintore, Nick R Price, Jacques R Chrétien, Alessandra Roncaglioni

**Affiliations:** 1 Food & Environment Research Agency, Sand Hutton, York, YO41 1LZ, UK; 2 BioChemics Consulting, 111 Bld. Duhamel du Monceau, 45166 OLIVET cedex, France; 3 Istituto di Ricerche Farmacologiche "Mario Negri", Via La Masa 19, 20156 Milano, Italy

## Abstract

**Background:**

The new European Regulation on chemical safety, REACH, (**R**egistration, **E**valuation, **A**uthorisation and **R**estriction of **CH**emical substances), is in the process of being implemented. Many chemicals used in industry require additional testing to comply with the REACH regulations. At the same time EU member states are attempting to reduce the number of animals used in experiments under the 3 Rs policy, (refining, reducing, and replacing the use of animals in laboratory procedures). Computational techniques such as QSAR have the potential to offer an alternative for generating REACH data. The FP6 project CAESAR was aimed at developing QSAR models for 5 key toxicological endpoints of which skin sensitisation was one.

**Results:**

This paper reports the development of two global QSAR models using two different computational approaches, which contribute to the hybrid model freely available online.

**Conclusions:**

The QSAR models for assessing skin sensitisation have been developed and tested under stringent quality criteria to fulfil the principles laid down by the OECD. The final models, accessible from CAESAR website, offer a robust and reliable method of assessing skin sensitisation for regulatory use.

## Background

The new European regulation on chemical safety, **R**egistration, **E**valuation, **A**uthorisation and **R**estriction of **CH**emical substances, (REACH Regulation 1907/2006) came in to effect from 1 June 2007. REACH, which is currently being implemented throughout member states, requires registration of all substances when produced and/or marketed in the EU in quantities exceeding 1 tonne per year, in preparations, or in articles. It has been estimated that over 30,000 chemicals used in industry will require partial or full safety testing to comply with the REACH regulations. At the same time, EU member states are committed to reducing the number of animals used in experiments under the 3 Rs policy (refining, reducing, and replacing the use of animals in laboratory procedures), and so considerable effort has been expended on searching for alternative methods of assessing the human and environmental safety of these compounds.

Computational techniques, such as those based on quantitative structure-activity relationship (QSAR) modelling, have the potential to offer one such alternative method. The lack of stringent quality criteria led to the development of many QSAR models in the past that lacked sufficient validation and reliability to make them adequate for regulatory use. Also until relatively recently the construction of QSARs was a highly variable process and required very sophisticated computer equipment. The rapid rise in desktop computing power has enabled relatively easy access to sophisticated applications in molecular modelling, computational chemistry, and machine learning, which have led to a burgeoning of the capability to produce QSARs for large and complex datasets. The formulation of the OECD Guidelines [1], further laid the foundation for a set of protocols for the development of robust and reliable QSARs intended for regulatory use.

The EU Research Project CAESAR [2] was responsible for developing robust QSARs for five toxicological endpoints of regulatory importance, one of which was skin sensitisation. A skin sensitiser is a substance that will induce an allergic response following skin contact. Substances are classed as skin sensitisers, if there is evidence in humans that the substance can induce sensitisation by skin contact in a substantial number of persons, or where there are positive results from an appropriate animal test.

Under REACH Annex VII, the sensitising potential needs to be assessed for chemicals produced or imported into the EU above the 1 tonne/year threshold. According to EC data, testing for skin sensitisation accounts for over 5% of the total use of commercial animal tests [3]. The OECD test guidelines 406 recommend the use of 30 guinea pigs for the Guinea Pig Maximisation Test or the Buehler Test [4], whilst OECD test guidelines 429 recommend the use of 25 mice for the local lymph node (LLNA) assay, [5]. Clearly one or more validated QSARs for skin sensitisation would be of considerable benefit both under REACH and the 3 R policies.

This paper reports the construction of global QSAR models obtained on a single dataset of 209 heterogeneous compounds using two computational techniques, particularly suited for data presented as classes of response.

## Results and discussion

### Adaptive Fuzzy Partition (AFP) models

From the original 502 calculated descriptors, 7 orthogonal descriptors remained in the models after reduction of descriptor space as shown in Table 1.

**Table 1:** The 7 descriptors remaining after reduction of descriptor space with HSA and used in the AFP models

*Symbol*	*Definition*
NN	Number of Nitrogen atoms
Gnar	Narumi geometric topological index
X2v	valence connectivity index chi-2
Eeig10r	Eigenvalue 10 from edge adj. matrix weighted by resonance integrals
GGI8	topological charge index of order 8
nCconj	number of non-aromatic conjugated C(sp2)
O-058	= O (atom-centred fragments)

Using the binary classifier, (NC = non-sensitiser, Sensitisers = weak/moderate/strong/extreme), the best model obtained by AFP was derived from the 7 descriptors computed with Dragon software and selected by HSA as described in the experimental section.

The results associated with the classification model established by AFP are shown in Table 2.

**Table 2:** Validation statistics derived from the best AFP model established on 42 non-sensitisers and 167 sensitisers using 7 DRAGON descriptors

	Training (%)	Test (%)
Accuracy	93	90
Precision	95	94
Sensitivity (class S)	96	100
Specificity (class NC)	79	75
F-measure	96	97

The best model is able to predict the experimental toxicity of the test set compounds with an accuracy of 90%. Moreover, this score is very similar to that associated with the training set prediction (93%), so the underlying feature of the model is not only predictive but also general. The robustness of this AFP model is mainly confirmed by the cross-validation score that is 77%.

Two compounds, outside the applicability domain as judged by the AFP software were unpredicted in the test set, and were suppressed for the calculation of the statistics parameters.

The statistics used in the table are derived as follows:

True positive (TP) = number of sensitisers correctly predicted

False positive (FP) = number of non sensitisers predicted as sensitisers

True negative (TN) = number of non sensitisers correctly predicted

False negative (FN) = number of sensitisers predicted as non sensitisers

Accuracy = (TN+TP)/(TN+FN+FP + TP)

Precision = TP/(TP+FP)

Sensitivity (true positive rate) = TP/TP+FN

Specificity (true negative rate) = TN/TN+FP

F-measure = 2/(1/precision)+(1/sensitivity)

It should be noted that the sensitiser class was the best predicted, with a validation rate of 94%. This result is very important for real applications of these models, because predicting a non sensitiser as a sensitiser (i.e. a false positive) is more acceptable than predicting a sensitiser as a non sensitiser (i.e. a false negative).

### Multilayer Perceptron (MLP) models

In order to provide more balanced dataset we also looked at binary classification models using the same data but incorporating the "weak sensitisers" in with the "NC" compounds in the "non-sensitiser" category. This gave a dataset with 108 non-sensitisers and 101 sensitisers, (moderate/strong/extreme categories; EC3 values >10).

Descriptor selection using cross correlation and co linearity routines resulted in 7 retained descriptors (Table 3).

**Table 3:** DRAGON Descriptors obtained by cross-correlation and multicolinearity in ADMEWORKS Modelbuilder, and used in the MLP models

*Symbol*	*Definition*
Me	mean atomic Sanderson electronegativity (scaled on Carbon atom)
PW2	path/walk 2 - Randic shape index
PW3	path/walk 3 - Randic shape index
PCR	ratio of multiple path count over path count
X3Av	average valence connectivity index chi-3
AAC	mean information index on atomic composition
IVDE	mean information content on the vertex degree equality

The descriptors, although different from those selected by the HSA routine are also a mixture of topological, connectivity and reactivity based descriptors, though interestingly the descriptors used are not directly correlated with those used for AFP modelling.

The results of the best MLP model are shown in Table 4.

**Table 4:** Validation statistics derived from the best MLP model of 108 non-sensitisers and 101 sensitisers using 7 DRAGON descriptors.

	Training (%)	Test (%)
Accuracy	84	71
Precision	80	76
Sensitivity (class S)	85	70
Specificity (class NC)	87	67
F-measure	82	73

Although the statistics for this model are slightly inferior to the AFP models, this model contains all the compounds in the set with no outliers excluded from the statistics and all test set compounds have a predicted value. The true negative rate is higher at 87% than the 79% of the AFP model possibly reflecting the significantly greater number of non-sensitisers in the dataset.

The Gerberick dataset has been widely used for building QSARs. These studies have recently been reviewed by Patlewicz and Worth [6]. Both global and local QSAR models for skin sensitisation have been developed. A global model is where the dataset is very structurally diverse and thus will reflect a range of different mechanistic actions among members of the set. Statistical methods are used to attempt to establish structure/activity patterns which transcend the mechanistic differences. However such models tend to have relatively low predictivity even when the statistics demonstrate that they are robust models. Local models are based on chemical similarity amongst the dataset which may be structural or mechanistic. Considerable work has been done on the mechanistic categorisation of skin sensitivity datasets, and this has been applied to the Gerberick set within the CAESAR project and has been published elsewhere [7]. One of the problems with local models is finding enough compounds that fall into the similarity classes to make statistically robust models, though categorisation can be useful in predicting sensitisation by read across.

Many of the QSARs reviewed by Patlewicz and Worth, do not meet the current standards of rigour required for regulatory use as laid down in the OECD Principles [1], and many others were not conducted with such utility in mind. The studies reported here under the CAESAR project [2] were conducted with a view to their use in REACH and thus with the OECD principles in mind. The skin sensitisation is a well defined endpoint of specific utility within the REACH framework. Both the AFP model and the MLP models use unambiguous algorithms whose input parameters are reported here and whose operation can be repeated by other users. Indeed the AFP model is now freely available to use via the CAESAR website [2].

It is difficult with global models to prescribe the domain in the context of particular chemical groups for which the model is valid. It has become common instead to define the domain in terms of the descriptor space of the training set. In its simplest form this is taken as the minima and maxima for each descriptor in the training set, though more complex domain calculations are often used. The descriptor space of the data set for each of the two models is shown in Table 5. Clearly compounds whose descriptor values fall outside those shown should not be used in the respective models. However it is interesting to note that although a small number of the test set compounds fall just outside the domain for one or more descriptors, it is not these compounds which the models fail to predict accurately.

**Table 5:** Descriptor Space Domain of the Dataset

Descriptor	Training Min	Training Max	Test Min	Test Max
Nn	0	5	0	8
GNar	1.3	2.3	1.0	2.9
X2v	0	9.97	0.27	10.0
EEig10r	-1.0	2.85	-1.01	1.74
GGI8	0.0	0.95	0.0	0.198
nCconj	0	6	0	6
O-058	0	6	0	3

				

Me	0.96	1.15	0.96	1.1
PW2	0.4	0.7	0.0	0.7
PW3	0.0	0.44	0.0	0.37
PCR	1.0	1.7	1.0	1.53
X3Av	0.0	0.52	0.0	0.36
AAC	0.88	2.10	1.05	2.25
IVDE	0.0	1.9	0.0	1.84

Similarly a mechanistic interpretation of global models is more problematic than that of local models, due to the diverse nature of the compounds in the dataset. Moreover, for an "in-vivo" endpoint, a number of different physiological and biochemical mechanisms may be at play in determining the final outcome of a particular compound. Again some limited mechanistic insight can be gained from an examination of the descriptors selected for the model. In both the AFP and the MLP models a number of molecular graph theory descriptors were used. Whilst such indices give a simplified index of the 2-dimensional structure of a compound they also have a direct relationship to reactivity and along with atomic and group-based descriptors (also used in our models), some link to mechanistic function can be inferred, and their utility has been well described in the literature [8]. Atomic characteristics (number of nitrogen atoms and number of non-aromatic conjugated Cp2 carbon atoms, in the case of the AFP model) have a direct relationship to functional groupings and hence molecular action, whilst electronegativity, (in the case of the MLP model) is a determinant in reactivity and thus has a direct bearing on mechanistic factors.

Both models were subject to rigorous validation procedures and the ultimate test being the use of test sets to determine the predictive power of the models. The final models, accessible from the CAESAR website [2], offer a robust and reliable method of assessing skin sensitisation for regulatory use.

The work presented here differs from other work on this dataset in a number of respects. The dataset has been more rigorously quality controlled and all efforts have been made to ensure issues such as isomerism, and structural veracity have been accurately assigned. Indeed we discovered that two of the compounds in the database were identical though named differently and were reported as having very different EC3 values. Any doubts concerning structures resulted in omission from our dataset. In addition we attempted to derive a more balanced dataset in some of the MLP modelling by including in the very small number of non-sensitisers, those that were classified as weak sensitisers from the original dataset.

The modelling methods chosen are particularly suited to classification problems of the type reported here. The AFP method has been demonstrated to perform well in toxicological classification problems [9,10] and when an MLP is correctly parameterised it can achieve the performance of maximum *a posteriori *receiver, which is optimal for classification problems.

Although many QSAR studies have been made on skin sensitisation datasets, as far as we were able to ascertain this is the first report of the use of AFP and MLP on LLNA data, though recently QMRF Number:Q16-10-22-170 http://qsardb.jrc.it/qmrf/ has been submitted to the JRC database in which another LLNA dataset has been analysed using MLP with similar results to our model though using quantum chemical descriptors.

The results of these studies indicate that QSAR skin sensitisation classification models using a fairly small number of simple descriptors can be constructed using either AFP or MLP, which will have good predictive power, especially for those compounds with some degree of sensitisation potential. The models are robust enough for regulatory use, though as with all such models should be used in conjunction with other approaches to develop a "weight of evidence" basis for conclusive decision making. Our models seem to have good predictive power across a wide range of chemical structural types within the descriptor space domain of the training set.

## Experimental

### Dataset

The dataset used for building QSAR models includes 209 compounds from the original 211 dataset of Gerberick *et al*. [11]. The data includes experimental values for the EC3, (the concentration of chemical that elicits a stimulation index of 3) from the Local Lymph Node Assay, (LLNA). The numerical data has been grouped into sensitiser classes according to convention as follows: Extreme sensitiser (EC3<0.1%), Strong sensitiser (0.1%<1%), Moderate sensitiser (1%<10%) and Weak sensitiser (EC3>10%). The use of data classes is of particular relevance for QSARs based on LLNA data because of the known intrinsic variability of LLNA data due to biological variation [6].

For each of the compounds, a 2D chemical structure was generated from the SMILES (Simplified Molecular Input Line Entry Specification) notation using Chemfinder [12]. Experience in the CAESAR project has shown that structural databases in the literature often contain errors or ambiguities so a thorough cross checking of chemical structures was carried out by two or more partner institutions. A number of incorrect chemical structures and ambiguities relating to isomerism were identified and corrected. Where ambiguities could not be resolved, those compounds were omitted from the dataset. After final checking, 209 compounds (out of a total of 211) remained in the dataset. The dataset was divided into training and test sets by random but stratified, sampling of 20% of the total dataset, giving 167 compounds in the training sets and 42 in the test sets, (see below). Adaptive Fuzzy Partition (AFP) modelling used a binary split of data, (sensitisers and non-sensitisers) in which only those compounds classed as "NC" in the original data were deemed to be non-sensitisers. For the multi-layer perceptron (MLP) modelling, non-sensitisers also included those compounds that were originally classified as weak sensitisers, (Table 6). Only the training set was used in the construction of the models, whilst the test set was reserved for validation of the models.

**Table 6:** Subdivisions of the skin sensitisation data set into binary classes.

	LLNA Class	Total compounds	Training	Test
Non-sensitisers	NC	42	34	8
Sensitisers	Weak+Moderate+Strong+Extreme	167	133	34

				

Non-sensitisers	Weak+NC	108	87	21
Sensitisers	Moderate+Strong+Extreme	101	80	21

		**209**	**167**	**42**

### Adaptive Fuzzy Partition, (AFP) models

502 molecular descriptors for 209 compounds were calculated on two dimensional structures using DRAGON professional 5.4 software [Talete srl, Milan]. They include (a) constitutional descriptors (atom and group counts); (b) functional groups, atom centered fragments; (c) topological, BCUTs, walk and path counts, autocorrelations, connectivity indices, information indices, topological charge indices, and eigenvalue-based indices.

#### Descriptor selection

To develop robust and reliable models the descriptor space was reduced by extracting the most significant variables. All variables were normalized into -1+1 range and variable selection was performed with a hybrid selection algorithm (HSA). This method combines a genetic algorithm (GA) with a stepwise regression [13]. Usually GA is applicable to problems where little information is available but it is not particularly suitable for local search. Therefore a stepwise approach was combined with GA in order to reach local convergence as it is quick and adapted to find solutions in "promising" areas already identified. To prevent over-fitting and a poor generalization, a cross validation procedure was included in the algorithm during the selection procedure. Thus, the dataset was randomly divided into training and validation sets in such a way that the fitness score of each chromosome was derived from the combination of the scores of the training and validation sets.

The following parameters were used in the data processing of the sensitisation data set:

*fuzzy parameters*: weighting coefficient was set equal to 1.5, tolerance convergence was equal to 0.001, number of iterations was 30 and cluster number was 6;

*genetic parameters*: chromosome number used was 10, chromosome size was equal to the total number of descriptors used; initial active descriptors in each chromosome was 8, crossover point number was 1, percentage of rejections was set at 0.1, percentage of crossover was 0.8, percentage of mutation was 0.05, number of generations was set at 10;

*stepwise parameters*: ascending coefficient was 0.02, descending coefficient was -0.02.

#### AFP algorithm

An in-house implementation of AFP was used to generate models able to predict the sensitivity class. This method is a supervised classification method implementing a fuzzy partition algorithm [14]. The method has been validated in data mining studies of a range of biological phenomena including toxicity prediction [9,10].

The AFP model for skin sensitisation was built on the training set by using the following parameters: maximal number of rules for each chemical activity = 30; minimal number of compounds for a given rule = 2; maximal number of cuts for each axis = 5. The trapezoidal parameters used were: p/w_i _= 1.25 and q/w_i _= 0.45.

The AFP method allocates degrees of membership of the different classes for each compound within a 0 to 1 range. Then, a compound is attributed to a given class if its degree of membership is greater than 0.5. The percentage of compounds correctly predicted is computed by comparing their experimental and predicted classes.

### Multilayer Perceptron (MLP), neural network models

A multilayer perceptron (MLP) is a feed forward network of neurons called perceptrons. The perceptron computes a single output from multiple real-valued inputs by forming a linear combination according to its input weights and then generating the output through a nonlinear activation function. A typical MLP network consists of a set of source nodes forming the input layer, one or more hidden layers of computation nodes between the input and output nodes, and an output layer of nodes [15]

MLP modeling was applied to the binary class data in which both non-sensitisers and weak sensitisers were classified as inactive (Table 6). The same starting set of 502 descriptors was used as in the AFP modelling.

#### Descriptor selection

Descriptor selection was carried out using ADMEWORKS Modelbuilder (FQS Poland), using a combination of cross correlation and co linearity routines.

#### MLP algorithm

Models were constructed using the MLP formalism in Neurosolutions (NeuroDimensions Inc, USA). The neural network consisted of one hidden layer and the number of processing elements (neurons) was chosen using the built in genetic algorithm routine. The algorithm determines the number of processing elements that produces the lowest cross validation error during a large number of training runs.

The transfer function used was the TanhAxon non linear axon. NeuroSolutions automatically scales and shifts the input data to match the range of the hidden layer's transfer function. In the case of the TanhAxon, the input data is scaled and shifted to lie between -1 and 1. The learning rule used was "momentum". The training was carried out over 1000 epochs. The cross validation option was selected in which the training stops when the cross validation error begins to increase. The best weights of the network are saved when the cross validation error is at its lowest point and are loaded into the network before the test set data is presented to the trained network.

## Competing interests

The authors declare that they have no competing interests.

## Authors' contributions

NRP, QC and JC developed and tested the MLP models. NP, MP, JRC developed and tested the AFP models. AR carried out quality assurance on chemical structures.

We acknowledge the EC funded CAESAR project. We thank in particular the following colleagues: Prof M. Cronin, Dr S. Enoch and Dr. J. Madden at LJMU, Liverpool, UK for collaboration; and Dr R. Kuhne, UFZ, Leipzig, Germany, for splitting of data set into training and validation sets.
